# 

**Published:** 1899-04

**Authors:** 


### NOTICES.

### The Northern Ohio Dental Association.

The 40th annual meeting of the Northern Ohio Dental Association will be held May 16th, 17th and 18th, 1899, in the Colonial Hotel, Cleveland, Ohio. A cordial invitation is extended to the profession.

W. JACKMAN, *Secretary.*
L. P. BETHEL, *President.*

Arrangements have been made for a very good meeting of this society. The programe is a very inviting one indeed, and contains a number of interesting subjects upon which papers will be read and discussions had. This society has grown to such proportions as to have great influence upon the profession in the northern half of the State.

It is to be hoped there will be a large attendance on that occasion of not only the membership, but by those who have not hitherto been members, and let all go prepared to add something to the interest of the occasion.

### EDITORIAL.

### THE DENTAL REGISTER,

A MONTHLY MAGAZINE DEVOTED TO THE INTERESTS OF THE

### DENTAL PROFESSION.

*Terms Reduced to* $2.00 *Per Year. Single Copies,* 25 *Cents.*

### EDITED BY PROF. J. TAFT, D.D.S.

—AND—

### Published by SAM'L A. CROCKER & CO., Ohio Dental and Surgical Depot.

The volume commences in January and closes in December.

The REGISTER being issued monthly is always fresh, therefore Indispensable to the practitioner who desires to keep posted up with the new inventions and improvements which are daily developed. Neither expense nor effort will be spared to give value and variety to its contents. Articles will be illustrated with well executed engravings whenever they will add to the interest or assist to explanation.

Its extensive circulation and frequency of issue make it one of the BEST DENTAL ADVERTISING MEDIUMS in the United States.

All subscriptions must begin with January or July.

Local or special notices, five lines or less, will be inserted for $3 each insertion; over five lines, 50 cts. per line.

All advertising bills payable in advance, or at furthest, after the issue of the first number containing the advertisement. All transient advertisements to be paid for in advance.

☞CONTRIBUTIONS SOLICITED.

Exclusively Editorial Communications should be addressed to the Editor.

The latest number of the REGISTER may be ordered with or without other goods at any time, and all letters should be addressed to [illegible]

